# Ideology and disease identity: the politics of rickets, 1929–1982

**DOI:** 10.1136/medhum-2013-010400

**Published:** 2013-08-05

**Authors:** Roberta Bivins

**Keywords:** Health policy, Politics, Public health, Nutrition and metabolism

## Abstract

How can we assess the reciprocal impacts of politics and medicine in the contemporary period? Using the example of rickets in twentieth century Britain, I will explore the ways in which a preventable, curable non-infectious disease came to have enormous political significance, first as a symbol of socioeconomic inequality, then as evidence of racial and ethnic health disparities. Between the 1920s and 1980s, clinicians, researchers, health workers, members of Parliament and later Britain's growing South Asian ethnic communities repeatedly confronted the British state with evidence of persistent nutritional deficiency among the British poor and British Asians. Drawing on bitter memories of the ‘Hungry Thirties’, postwar rickets—so often described as a ‘Victorian’ disease—became a high-profile sign of what was variously constructed as a failure of the Welfare State; or of the political parties charged with its protection; or of ethnically Asian migrants and their descendants to adapt to British life and norms. Here I will argue that rickets prompted such consternation not because of its severity, the cost of its treatment, or even its prevalence; but because of the ease with which it was politicised. I will explore the ways in which this condition was envisioned, defined and addressed as Britain moved from the postwar consensus to Thatcherism, and as Britain's diverse South Asian communities developed from migrant enclaves to settled multigenerational ethnic communities.

‘[T]o make … the welfare foods a luxury is bound slowly to undermine the fine achievements in the field of preventive medicine. … It will be an insidious process, and only the social historians will be able to point to the folly of this action.’ Baroness Edith Summerskill, 1961^1^

In the last two decades, media reports addressing the apparently rising incidence of a childhood disease, nutritional rickets, have increased in number and in frequency. While rickets undoubtedly persists, rates have remained vanishingly low in the global North. Nonetheless, reporters and clinicians typically express shock or horror at finding any cases at all of this well-understood, readily prevented and easily cured condition. Such expostulations—like the condition which provokes them—are consistently framed in terms of ‘history’: headlines describe the return of a ‘Victorian’ disease, one associated with extreme poverty in the past. And certainly, perceptions of rickets have deep historical roots, particularly in the UK. Rickets was first formally characterised in 1645 in a thesis that specifically designated it as the ‘peculiar and domestic scourge to our English infants’ for its strong association with Britain's gloomy skies and dark winters.^2^ ^3^

In the USA, where rickets has been largely controlled through the voluntary commercial fortification of most liquid whole milk and many breakfast cereals, contemporary media attention prompted the Centers for Disease Control to issue a press briefing on the disease in March, 2001; to convene an expert panel on vitamin D in October of the same year; and to repeatedly issue guidance advising supplementation for specific vulnerable groups.^4–6^ Combined with suggestive data that vitamin D sufficiency, well-known for its protective and curative effects on rickets, might also guard against diseases as varied as cancer and hypertension, these actions stimulated pressure for research. In 2010, the CDC's ‘Public Health Grand Rounds’ series sifted the data produced by the annual National Health and Nutrition Examination Survey (which specifically monitored vitamin D status since 1988) to elicit information about patterns of D deficiency, while experts at the National Institutes of Health called for action to improve assessments of vitamin D in foods and vitamin D status of individuals.^7^ In Britain, media interest has been similarly common, though official attention was slower to follow (a working group was not established until 2011).^8^ Analysis of an earlier rickets ‘crisis’ will shed light on its contemporary resonance, and Britain's sluggish response.

From the 17th century to the 20th, rickets was a feature of life in Britain's industrial cities; its prevalence encouraged nosological speculation and therapeutic experimentation. By the 1920s, dueling research teams had shown that sunlight and cod liver oil could prevent or cure the condition.^9^ Their bitter dispute over whether rickets was an environmental or a nutritional disease would echo in heated political debates about the recurrence of rickets in postwar Britain. It is on these debates that I will focus here, to assess the impact of politicisation (and later racialisation) on conceptions and public health responses to curable chronic morbidity in a modern welfare state. What is the value of disease to politics, and conversely, how can political interest(s) stimulate reinterpretations of disease?

While a range of genetic disorders and drug interactions can cause rickets and osteomalacia, the most controversial cases in postwar Britain, as in the USA, derive from simple vitamin D deficiency. The body's requirements for vitamin D (actually a secosteroid) can be met in a range of ways; in 20th century Europe, diet, food fortification, specific supplementation and endogenous synthesis of D in ultraviolet-exposed skin have all played key roles. Well known for their protective effects since the 1910s, natural and artificial ultraviolet light and vitamin D-rich foods like cod liver oil became the primary tools in interwar British campaigns to reduce the incidence of rickets among the urban poor. However, vitamin D deficiency rickets persisted as a familiar disease of working class childhood until it was virtually eradicated by the population feeding and supplementation programmes of World War II.

After the war, Britain's battered medical infrastructure and strained public health services faced significant challenges. The new National Health Service (NHS) elevated public expectations already raised by reports of wartime ‘medical miracles’ (notably the advent of antibiotics). However it opened in the middle of a national economic crisis; faced surging demand rooted in the long-unmet health needs of those previously unable to afford medical care; was encumbered by a complicated tripartite structure separating preventive and curative medicine; and operated from one of the weakest ministries in Whitehall. No longer facing the exigencies of war, national governments offered great rhetorical support to the NHS, but systematically reduced the proportion of each annual budget devoted to disease prevention, health promotion and other non-hospital health services.^10^ Nonetheless, the NHS rapidly became a much-loved and much-lauded feature of Britain's welfare state, emblematic of postwar egalitarianism and of British modernity. The victory over rickets, like the rapid declines in tuberculosis incidence, represented to many an end of ‘slumdom’ and the brutal social inequalities which had previously allowed this easily prevented and cheaply cured disease to thrive.

## Rickets and modernity

While rickets had been an expected penalty of poverty in the early 20th century, it became a sensitive political issue during the ‘Hungry Thirties’. Governmental indifference to the unmet nutritional needs of working class families in a period of economic depression and skyrocketing unemployment triggered an extensive critical literature, from Allan Hutt's 1933 excoriation *The Condition of the Working Class in England* to George Orwell's 1937 *The Road to Wigan Pier*.^11^ ^12^ Ministry of Health optimism about improving population health likewise contrasted sharply with a growing independent literature pointing out appalling health disparities between rich and poor in areas like neonatal, postneonatal and maternal mortality and morbidity. Meanwhile, new diagnostic techniques were replacing the unassisted ‘clinical gaze’, revealing dismal levels of chronic and subclinical disease among urban children.^13^ As an emblematic indicator of preventable child malnutrition, rickets was the focus of particular attention and appeared regularly in parliamentary debates.^14–16^

Many medical experts concurred broadly with Ministry of Health optimism; for instance, royal physician Thomas Jeeves Horder insisted in 1937 that rickets was ‘fast dying out.’^17^ However, studies reporting that over 80% of children in London and Durham showed symptoms of the condition contrasted sharply with the rosy official picture, attracting commentary in the press and Parliament.^16–21^ The intense attention paid by Britain's wartime planners to preventing rickets suggests its totemic status.^22^ ^23^ So too does Winston Churchill's insistence on the recall and destruction of Abram Games’ famous ‘Your Britain: Fight for It Now’ propaganda poster contrasting an image of the Finsbury Health Centre with a ruined playground haunted by a rickety child (viewable at http://www.iwm.org.uk/collections/item/object/10300 (accessed 30 March 2013)).^24^ ^25^ Thus it is unsurprising that rickets, and fears of its resurgence, transfixed the medical researchers and bureaucrats charged with managing Britain's food supply during World War II.^26–30^

The details of Britain's wartime nutrition strategy are well-known; in relation to the decline and near-disappearance of rickets, three aspects were crucial. First, rationing and state-run canteens improved access to a sufficient and nutritious diet for the poor. Second, by mandating the exclusive milling of bulky, nutrient-rich (but unpopular) high-extraction flour, and by fortifying flour with calcium, and margarine with vitamins D and A, the state ensured national dietary adequacy in key nutrients. Finally, expectant and nursing mothers, infants and young children (and other ‘vulnerable’ groups) received special diets and supplements, including further vitamin D.^31^ ^32^ In addition, the war-time government gave nutritional education unprecedented support, weaving advice about diet, health and nutrition into countless aspects of its wider propaganda programmes.^33^ By the time rationing ended in 1954, voices from across the public sphere were ready to declare victory over rickets (along with other nutritional deficiency diseases). Nobel prize-winning physiologist Sir Henry Dale happily announced that rickets had been ‘practically wiped out’.^34^ The left-leaning *Manchester Guardian* newspaper likewise cheered the disappearance of ‘rickets cases and other symptoms of the slums’.^35^ In succeeding decades, rickets was conventionally described as a vanishing, vanquished or historic disease, at least for the majority population.^36–38^

In Parliament, rickets attracted extravagant rhetorical flourishes; its eradication was equated with Britain's scientific and social modernity, while any signs of resurgence provoked outcry. Thus, politicians declared the elimination of rickets to be the ‘most spectacular change which has taken place in this country’.^39^ Similarly, the inability of staff at a major hospital to find a single teaching case of rickets evidenced the ‘infinitely better’ health of the community at large.^40^ In contrast, an apparent increase in rickets in the early 1960s was decried as ‘deplorable at this stage of medical knowledge’.^41^ Indeed, as one parliamentarian observed in 1946, with medical knowledge of rickets ‘practically complete’, it had become a governmental responsibility to ensure that rickets in Britain remained rare. 'Rickets’, he concluded, had ‘ceased to be a medical disease and become a political one.’^42^

## The hypercalcaemia crisis

The state's task of controlling rickets was hampered by the 1955 discovery of an unwanted side effect of governmental supplementation and fortification policies. Reports emerged about a seemingly new nutritional disorder, hypercalcaemia. Apparently caused by an excess of vitamin D, high levels of calcium in the blood of affected infants and children were linked to a dangerous array of symptoms ranging from irritability and loss of appetite to dehydration, weakness and convulsions.^43^ Appalled that the feted wartime regime of high fortification, free supplementation and rationing might be causing iatrogenic illness, the Ministry moved swiftly to reduce the levels of mandatory fortification, and to lower recommended levels of supplementation.^44^ ^45^ Where wartime food policy strove to eradicate rickets from Britain, nutrition policy post hypercalcaemia set a much lower (but purportedly safer) defensive goal: to protect most UK children from rickets, while exposing as few as possible to hypercalcaemia.

In practice, medical civil servants and politicians proved willing to risk an increase in rickets—a curable disease that could be blamed on bad parenting rather than bad policy-making—to avoid accusations of iatrogenesis. From 1957, therefore, mandatory fortification was retained only in the foods commonly consumed by the most vulnerable Britons: infants and the poor. The state required the fortification, at a new lower level, of only three foods: margarine (vitamins A and D), white flour (calcium, iron, B1 and B3 —all removed in the milling process), and infant feeds (a comprehensive array of added micronutrients). Otherwise, the state was (unenthusiastically) permissive of manufacturer fortification. Unlike the USA, in Britain the fortification of liquid whole milk never became a commercial norm.^46^ This was largely due to legislative protection of milk purity—but governmental squeamishness in the face of long-running public hostility to mass health mandates also played a role (see below).

Within the Ministry, fortification was badly tarnished by this episode; it would not regain bureaucratic favour for generations (indeed, despite strenuous medical lobbying, no new fortification mandates have been introduced since the 1940s). Designed to barely protect only vulnerable members of the majority community, this policy would have unexpected implications for the health of Britain's ethnic minorities, and in turn for the health of race relations in the medical sphere. It meshed perfectly, however, with the broad trend of government policy to withdraw from regulation and control in the area of nutrition, and to intervene in the national diet only via health education and (doctor-led) individual supplementation.

## Rickets returns

After 1954, successive conservative governments gradually shrugged off the accumulated responsibilities for population feeding taken on in the light of the politically charged context of the ‘Hungry Thirties’ and increased by rationing. Since each move to diminish such interventions triggered political resistance from the Left, it became imperative for the Ministry of Health to develop evidence that governmental intervention in British diet was no longer necessary.^47^ A series of parliamentary questions and debates in the late 1950s exposed significant apprehension about the systematic reduction of food subsidies intended to ensure nutritional equity between rich and poor.^48^ ^49^ A second wave of changes in 1961, raising the costs of Welfare Foods to all but the poorest families, stirred similar anxieties and bad publicity. Speakers on both sides of the Commons feared a resurgence of rickets, particularly as evidence accrued of a decline in consumption of Welfare cod liver oil after a charge was introduced for the supplement.^50^ ^i^ Once again, as in the 1930s and late 1950s, the government stood accused of economising to the detriment of expectant mothers and young children through what opponents called the ‘meanest, the most miserable and the most despicable’ of all its proposed cuts.^51^ Only months after the 1961 cuts came into effect, some areas began to report the reappearance of rickets among UK children.

Scathing political critique spurred the Ministry to investigate the nutritional health of the nation's children via a British Paediatric Association member survey.^52^ At this point, the Ministry of Health was interested in the national effects of food policy on Britain's majority population. It was, after all, initial reports of rickets among Scottish children that triggered consternation.^53–56^ However, the British Paediatric Association drew attention to the impact of the new Commonwealth immigrants on the incidence of malnutrition in Britain. For them, ‘Large scale immigration … of families from the Commonwealth’ explicitly ‘caused’ the apparent increase in nutritional disorders. Their survey therefore sought information about affected children's race, place of birth and date of entry to the UK.^57^ In describing immigration as *causing* rickets, these documents also foreshadow what would become an established line of departmental argument in the 1970s, when ‘Asian rickets’ was constructed (like tuberculosis (TB) before it) as an imported illness, rather than a consequence of migrants’ poor housing and deprived urban environments.

Reports soon emerged confirming the higher rates of rickets among recent migrants to Britain from the ‘New Commonwealth’ (ie, from Britain's former tropical colonies), and its presence among the urban poor more generally.^56^ ^58^ ^59^ However, interpretations of this association varied widely between those who saw the conditions as imported (either as pre-existing malnutrition or through the continuation of inappropriate cultural practices) and those who regarded them as evidencing the poor conditions in which the newcomers were trapped. Others, particularly those steeped in the discourses of social medicine, argued that a far wider segment of Britain's children were affected and deserved state protection in the form of continued welfare feeding. Finally, there were those, especially in the Ministry of Health (from 1968, the Department of Health and Social Services, (DHSS)) itself, who scoffed at burgeoning concerns about nutrition. WTC Berry, who led the Ministry's nutrition unit treated diagnoses of rickets with particular suspicion. As he grumbled, ‘Since rickets at least is usually subjectively diagnosed it could be that any Clinic Medical Officers who are dissatisfied with the increased charge in the price of welfare foods might tend to overdiagnose.’^60^ Thus the ease and thoroughness with which rickets could be politicised rendered all diagnoses of the disease ‘suspect’, and may have blunted the policy response to its reappearance. Berry would later complain that rickets ‘excited great emotion’ not as a threat to public health, but as ‘a sign of social regression.’^61^

## Rickets and politics of poverty in the postwar consensus

Certainly by 1964, parliamentarians were engaged in political point-scoring over the reappearance of rickets in Britain. Other factors too raised the profile of rickets (and osteomalacia) over the course of the 1960s. Public and political discontent with levels of inmigration, and discomfort at increasingly evident signs that Britain was failing to model the ‘colour blind’ social values and responses that it had for so long urged on its ‘multiracial empire’ directed greater attention to all aspects of immigration and ‘race relations’.^61–64^ The health of non-white migrants in particular became a delicate political issue. Moreover, as I have argued elsewhere, the return of deprivational rickets (rickets unrelated to metabolic disorders) represented a significant opportunity for British biochemists and metabolic medicine, who eagerly capitalised on it, producing a stream of publications on rickets in the process.^65^ As new and ever more sensitive diagnostic techniques emerged for assessing the circulating levels of vitamin D in the body (and thus a given individual's nutritional status and risk of morbidity), medical opinion about the incidence and severity of vitamin D deficiency among British children and adolescents fragmented. While some commentators accepted only radiological evidence as proof of rickets, others were aghast at biochemical studies revealing widespread subnormal nutrition. As in the 1930s, in the absence of medical consensus, politics flourished, not least because more detailed studies of affected children and their families revealed the persistence of extreme social deprivation in some areas.^53^ ^66^

Calls for action in the 1960s relied primarily on claims that rickets was affecting the indigenous population. They were almost entirely ignored by the central health administration, precisely because the emerging rickets cases themselves did not directly validate the Opposition's political claims. Indeed, rather than unease, the results of early studies prompted relief at the Ministry of Health: whether local or newly arrived, the families affected by rickets were poor enough to qualify for free supplementation and welfare foods—thus exonerating the new charges introduced in 1961.^67^ Better still, in the eyes of a Ministry eager to avoid opening a second front in the battle over ‘mass medication’ (fluoridation of the water supply was already the subject of heated debate), all increases in rickets occurred in areas also marked by high rates of immigration.^68–70^ The Ministry of Health could therefore take refuge in the idea that rickets, like tuberculosis, was re-entering Britain by plane rather than by policy.^71^ Yet while this diminished the *political* saliency of the new rickets cases for some—the *BMJ* doubted that the new cases could ‘be reasonably blamed on the decision to reduce the intake of vitamin D’—others, including the British Medical Association, continued to insist on their medical and social significance.^72^

Medical professionals working in areas with large immigrant and ethnic communities urged greater attention not only to the recurrence of rickets, but also to the wider problems of undernutrition among the children of migrants, using dramatic graphs and images of bow-legged babies to emphasise the situation's urgency.^73^ Simultaneously, researchers from Glasgow challenged the comfortable assumption that ‘coloured immigrants are the problem’. This was, they claimed, the ‘soothing syrup’ of state complacency; any return of rickets instead demanded a return to the vigorous prophylaxis and education of the war years.^74^ Opposition politicians, too, were suspicious of the Government's efforts to blame immigration for the rise, and to clothe their decision in medical impartiality.^75^ Many accused the Conservatives of attacking the poor, and of failing to meet earlier promises to reassess the changes in welfare feeding should any signs of rickets subsequently emerge.^76^ ^77^ Labour, however, proved only marginally more willing to tackle rickets and the poor nutrition it presumptively signified when it became the party of government in October 1964. While promoting more detailed surveys of population nutrition and the prevalence of rickets, successive Labour administrations declined to restore levels of nutritional support.^78^

For the remainder of the decade, medical and media outlets sporadically returned to the subject of rickets. Interpretations and responses to the disease differed significantly depending on the population in which its return was feared. Rickets among recent immigrants was largely dismissed as a transient ‘disorder of transplantation’ caused by (maternal) ignorance rather than poverty.^79^ ^80^ Among the indigenous poor, however, it was seen as symptomatic of ‘a much deeper malaise’, reflecting widespread undernutrition.^81^ Their plight revealed ‘poverty, social incompetence and poor health marching together.’^82^ Thus, just as individual diagnoses of rickets were perceived to depend on the politics (and surveillance tools) of the diagnostician, so the political and social meanings of rickets depended on who observed it and whom it affected.

Overall, rickets had far less political resonance under Labour: untainted by the scandalous inequalities of the 1930s, Labour's failure to restore Welfare Food provision to postwar levels did not tarnish its reputation. In consequence, even when welfare feeding programmes faced further cuts, rising levels of rickets failed to attract the levels of political and media attention they had garnered under a Conservative government in the early 1960s. When a Labour government in 1968 removed the provision of school milk from children in secondary education, the gesture provoked open rebellion only from Labour's own Left wing. Labour Members of Parliament (MPs) fought the cut, using the by now customary rhetoric of governmental ‘meanness’ and dangers to the health of children.^83^ ^84^ But rickets itself, described under the Tories as ‘the most emotion charged of all the symptoms of deprivation’, loomed small in these debates.^82^ Only two speeches—both from Labour MPs—specifically mentioned the condition. Strikingly, both took for granted that the deficiency disease, like tuberculosis, had been despatched by the advent of the Welfare State. In a sign of what the *Times* described as the new and ‘virtually non-party’ acceptance of benefit-selectivity, Labour's proposed reduction in welfare feeding drew little response from the Conservatives, who shied away from mentioning rickets at all.^85^

In contrast, when Conservative Minister for Education Margaret Thatcher subsequently proposed further reductions in the provision of free school milk in 1971, rickets immediately regained its place at the heart of parliamentary debate. Once again, its supposed absence from contemporary Britain was a marker for the progress and modernity emergent from the equalising effects of the Welfare State. Jarrow MP Ernest Fernyhough, for example, rose to oppose the measure, cautioning: ‘It was the proud boast of this country in the middle 1950s that, because of our welfare and health services, British doctors had to go abroad to study rickets because none of our children were suffering from it.’ Changes in government policy implicitly threatened that progress, by risking the return of rickets.^86^ Other MPs worried about ‘putting the clock back two generations’ and the return of schools full of rickety children with their legs in irons.^87^ ^88^ MP Neil Kinnock condemned the policy at the Bill's second reading as a violation of the postwar consensus, equating fairly distributed nutrition with modern civilisation itself: ‘The public had come to accept that since the war a degree of natural justice, a feeling of compassion, had developed amongst people regardless of party … Now this atavistic Government have moved back to the priorities of a bygone age. … This is a barbarian Bill which is the product of a barbarian mind.’^89^ In effect, from the end of rationing to the end of the 1960s, rickets attracted political and public attention only when party politics—and the deeply embedded association of Tories with the ‘Hungry Thirties’—made it rhetorically valuable as the ultimate signifier of ideologically driven regression and social inequity. In these circumstances, rickets retained its identity as the ultimate ‘evidence of poverty’.^90^

Over the course of the 1960s, however, a new form of social inequity attracted increasing political, social and media attention: racial discrimination. As discourses of ‘race relations’ entered mainstream politics, rickets offered and became subject to new kinds of rhetorical traction. If the debates over school milk in 1971 revitalised the familiar association between rickets and economic inequality so vitiated under a Labour government, a 1973 exchange between Labour MP Laurie Pavitt and Margaret Thatcher signalled things to come. Drawing on press reports of a recent study in Birmingham, Pavitt questioned Thatcher, asking whether she was aware of the ‘epidemic of a new form of rickets, biochemical rickets’, symptoms of which had been found in up to 20 per cent of tested schoolchildren. Thatcher's rebuttal focused on two points. First, she raised the still-unresolved question of how rickets was to be diagnosed—whether through clinical signs, biochemical abnormality or radiological proof of bone malformation—and implied that only radiologically apparent disease constituted rickets. Building on this strict definition, she claimed that only ‘immigrant children’ in the Birmingham survey had displayed signs of rickets.^91^ Thatcher's public reframing of rickets as a disease of a transplanted minority rather than an indicator of poverty among the majority was intended to dilute the political impact of its reported rise. To a certain extent, her strategy proved effective. From 1973 to 1979, party-political interest in rickets declined, as evidenced by a sharp fall in references to the disease on the floor of either House.^ii^ However, in the wider public sphere, the changed identity of rickets led to a shift rather than a diminution of its perceived significance.

## Re-envisioning rickets, 1971–1979

Since the early 1960s, medical researchers and public health workers had documented the rising incidence of rickets and osteomalacia among the children of recent migrants from South Asia. This trickle of reports became a stream after the arrival of refugee families, also of South Asian descent, expelled from Kenya (1967–1968) and Uganda (1972).^92–95^ In the process, the once-familiar disease gained a new medical and popular identity: ‘Asian Rickets’.^96–103^ In turn, because any resurgence of rickets prompted some political commentary, these medical reports entered the mainstream media.^38^ ^104^ ^105^ Like rickets’ earlier and enduring identification as the ‘English disease’ and ‘scourge of poverty’, this new understanding of the disease was readily politicised.

As Thatcher's 1973 riposte suggests, the idea of an ‘Asian rickets’ was initially most useful to politicians eager to diminish the political impact of accusations that rickets had been revived in Britain by neoliberal cuts to population nutrition programmes—like Thatcher's infamous ‘milk-snatching’. Medical civil servants too used the concept to argue that the rise in rickets was a merely local problem, perhaps imported, and certainly best addressed by local health authorities rather than by the kinds of central action that had eliminated the disease during World War II.^106^ ^107^ However, the enthusiastic adoption of ‘Asian rickets’ by politicians and the DHSS had unintended consequences, consequences which made the ‘new’ form of rickets just as controversial as its predecessors by the end of the 1970s.

To understand the political impacts of rickets’ new identity, it is important to recognise the social and legal context into which ‘Asian rickets’ was introduced in the 1970s. In an attempt to balance what were internationally perceived as racially discriminatory changes to UK immigration law initiated by the 1962 Commonwealth Immigrants Act, Wilson's Labour government in the mid-1960s passed a succession of new laws intended to eradicate racial discrimination and promote equality within Britain. The largely toothless Race Relations Act of 1965 was strengthened and extended to employment, housing, and crucially, public services in 1968. Both Acts were replaced (during James Callaghan's Labour administration) by the Race Relations Act of 1976 and the creation of the Commission for Race Equality.^108^ These legislative innovations heightened sensitivity to potentially discriminatory practices and introduced legal sanctions intended to address ‘racial disadvantage’ by prohibiting indirect as well as direct racial discrimination. Indeed, some scholars have argued that they prompted a ‘rediscovery of race’ after an era of ‘colour-blind consensus’.^109^ They certainly provoked concern about the legality of previously common—and in some cases welcome and effective—public health and service interventions targeting specific minority groups.^110–114^ The Race Relations Acts also opened new pathways for activists and citizens to seek redress for failings in Britain's public services, and made examples of health disparities between the majority and ethnic minority communities far more newsworthy.

All of these factors played a role in rendering the incidence of rickets and osteomalacia among British Asians emblematic of wider concerns about racial equality and access to care. While their political masters lost interest in rickets as an emblem of socioeconomic disparity, the DHSS came under increasing pressure to take action against vitamin D deficiency among Asian communities as a signifier of racial inequality. Much of that pressure came from within the medical and biomedical research communities. Even medical insiders called for the Department to learn ‘from the history of vitamin D deficiency and rickets in Britain’, rather than continuing to pursue ‘improbable causes of Asian rickets and osteomalacia’ in the form of culturally influenced dietary or dress preferences.^115^ At a meeting of the Department's Working Party on Infant Foods, DHSS staff faced open criticism: ‘provocative remarks were made about rickets in the Asian community with the usual implication that the Department is not dealing adequately with the problem’.^116^ By 1978, the Department faced attacks even in the *Health and Social Services Journal*, the trade paper of the NHS and local health authorities. Under the emotive headline ‘What Kind of Welcome?’, journalist Ron McKay used rickets as ‘a perfect continuing example of the failure of the health community to treat the diseases and ailments of Britain's Asian population,’ and criticised the DHSS response—17 years of deliberations and delays—as ‘slow and incompetent in the extreme’.^117^ The broadsheet newspapers too played a key role, raising the profile of rickets among British Asians with headlines like ‘Rickets danger for Asians in Britain’ and ‘Aid Urged for Asians Affected by Rickets’. These drew attention to the marked difference between the Department's wartime response to rickets in the general population and its sluggishness in relation to ‘Asian rickets’.^118^ ^119^ But ultimately the DHSS—and more specifically, a newly installed Minister for Health, Gerard Vaughan—was driven to respond by the one medium they had consistently and explicitly refused to deploy in their attempts to educate British Asians about rickets prevention: television.

In 1979, Granada Television's ‘World In Action’ programme aired a critical segment on rickets among Britain's Asian communities. Vaughan declared it a ‘disaster’ for the Department, for both promoting fortification (recently rejected by the Committee on Medical Aspects of Food) and presenting the Department as ‘doing little’.^120^ To silence these criticisms, rickets and osteomalacia became the first ethnically associated illnesses to attract a targeted response from Britain's central health authorities in the postwar era. Vaughan personally initiated a new approach, inviting ‘community leaders’ join a Working Group on Rickets and to advise the Department as it formulated a new national campaign to ‘Stop Rickets’ among British Asians.^121^

Announcing his new venture to the House of Commons, Vaughan confirmed Thatcher's identification of rickets with ‘immigrants’ (ignoring a generation of British-born children of Asian heritage): ‘This condition was virtually eradicated until the late 1960s and the early 1970s. At the moment, it is almost entirely a problem of immigrants …’ However, the political meaning of that identification in 1980 differed significantly from its import in 1973. Instead of justifying public health inaction, it demanded active engagement with ‘leaders of the immigrant community’ and ‘members of immigrant groups’—who were themselves increasingly able to express dissent and dissatisfaction with DHSS approaches to rickets.^122^ As an internal memo admitted, Vaughan's initiative ‘started from the recognition that there was a need to explain to leaders of the Asian community the reasons why the Government was not proposing to recommend further fortification of foodstuffs with vitamin D (a move to combat rickets which has been widely canvassed).’^123^

Driven forward by Vaughan himself, the ‘Stop Rickets’ campaign was designed to showcase engagement with long-ignored communities, and simultaneously to demonstrate the cost-effective power of education-only campaigning as compared to ‘nanny-state’ regulation and ‘mass medication’. But it was more than just ideological show-boating. ‘Stop Rickets’ demonstrated for the first time a growing realisation that medical professionals and civil servants needed partners on the ground to effectively address problems of ethnic health. Less positively, it signalled the successful reinscription of rickets and osteomalacia as exclusively ‘Asian’ health concerns. This shift did not, as perhaps some politicians had hoped, entirely silence the political echoes of an easily prevented and readily cured nutritional deficiency disease returning to threaten the citizens of a modern welfare state. However, it did render them less audible in Parliament and in the public sphere. As a cheery *BMJ* ‘Letter from Westminster’ in 1982, ‘most people’ were ‘probably unaware’ of the Stop Rickets campaign altogether because it was so tightly focused on Asian communities.^124^

## Conclusion

To return to the question raised in the introduction, I would argue that rickets, since at least the 1960s, has received attention disproportionate to its incidence and severity precisely because of its symbolic value. For politicians and medical professionals eager to champion an active and progressive welfare state, and to preserve the postwar model of social medicine, rickets was a powerful lever with which to jolt their opponents. For British Asian communities, however, the politically motivated reinterpretation of rickets as an ‘Asian’ disease would prove a double-edged sword. While rickets’ symbolic significance created an opportunity for at least some Asian leaders and community members to engage directly with health policymaking within the DHSS, the Stop Rickets campaign also served as an effective distraction from larger health concerns affected Britain's Asian ethnic minorities. The campaign was seen as aiding the integration of Britain's Asian communities into the NHS, unmasking other significant health problems, and improving ‘race relations generally’; thus it alleviated pressure on the DHSS to dedicate more substantive efforts (and resources) to improving these communities’ access to NHS and other health services. Moreover, driven by the political necessity to ‘show that the Department was concerned’ about Britain's ethnic minorities, the campaign largely ignored the one point of general consensus that had emerged from the Department's decade of deliberations about the incidence and measurement of vitamin D deficiency: that osteomalacia was dangerously prevalent among the housebound elderly of all ethnic origins.^120^ No measures addressed this problem, further confirming the risk that a politicised—and racialised—disease identity can have on the assessment, measurement and protection of the public health.

Anxieties about a recrudescence of the ‘English disease’, with all its associations for the politics of welfare, overshadowed the incidence of rickets and osteomalacia among Asian ‘immigrants’ in the 1960s and early 1970s. Similarly, ‘Asian rickets’ and its implications for the politics of race displaced attention from persistent vitamin D insufficiency among the general population, and the well-recognised problem of osteomalacia among the elderly. Neither problem—nor indeed the continued prevalence of vitamin D deficiency among some parts of the British Asian population—has been resolved today. Today, as in the 1970s and 1980s, calls for fortification, wider supplementation and more active governmental engagement have produced little action. I suggest that this stasis does not merely evoke the past but rather reflects very similar political tensions, debates and ideologies. Like the potency of British sunshine, the political power of rickets may vary; however, like the disease itself, it has not disappeared entirely.

## Footnotes

i The 1957 changes had seen (among other things) prices of liquid and vitamin D fortified National Dried Milk rise, creating an incentive for the use of infant milk tokens to supply milk for household use, while shifting infants to cheap proprietary baby foods. In 1961, charges were introduced for the formerly free Welfare cod liver oil and vitamins A and D supplement tablets. Precipitous falls in uptake of these supplements followed.^46^

ii Where parliamentarians cited rickets 116 times between 1963 and 1973, the succeeding decade saw only 61 mentions (despite a cluster of self-congratulatory references to the ‘Stop Rickets’ campaign in 1981–1983). In the 5 years after Thatcher insisted that rickets was an ‘immigrant’ problem, the fall was even more precipitous, with the condition arising only 21 times in parliamentary debate.
